# Accidental Chlorine Gas Exposure in a Pediatric Patient: A Case Report

**DOI:** 10.5811/cpcem.2020.3.46746

**Published:** 2020-04-23

**Authors:** Ashley Antolick, Lindsey Ouellette, Bryan Judge, Brad Riley, JS Jones

**Affiliations:** Spectrum Health - Michigan State University Emergency Medicine Residency Program, Grand Rapids, Michigan

**Keywords:** Chlorine gas exposure, treatment, nebulized sodium bicarbonate, pediatric

## Abstract

**Introduction:**

Chlorine gas is a known irritant of the respiratory tract, which may cause damage to various systems depending on time of exposure and concentration of the gas. Current treatments are mainly supportive. While no definitive studies have been completed to date, it has been noted that treatment with a sodium bicarbonate solution via nebulizer may lead to improved outcomes for patients dealing with chlorine gas exposure.

**Case Report:**

We present a case of a nine-year-old child arriving at the emergency department after exposure to chlorine gas. Complete recovery from his symptoms occurred rapidly with the administration of nebulized sodium bicarbonate.

**Discussion:**

Inhaled chlorine gas acts as a mucous membrane irritant, with symptoms usually beginning within minutes of exposure. Inhaled nebulized sodium bicarbonate has been suggested as a therapy for chlorine exposure. Although its mechanism of action is not well understood, it is thought that inhaled sodium bicarbonate neutralizes the hydrochloric acid formed when the chlorine gas reacts with the water in the lungs.

**Conclusion:**

Nebulized sodium bicarbonate solution at a low concentration appeared to rapidly and effectively reverse the symptoms due to chlorine gas inhalation in a young child.

## INTRODUCTION

Chlorine gas exposure can occur from multiple sources including in-home cleaning products or accidental mixing of ammonia and bleach, swimming pool chlorination reactions, industrial accidents, and intentional exposures with intent to harm as a chemical weapon.^1^,^2^ Because of the prolific and widespread use of chlorine in both industrial and household environments, the gas is one of the most common substances involved in toxic inhalation.^3^ Toxic effects are dependent on the time of exposure and the concentration of the gas and are mediated by chlorine’s reaction with water on the mucosal surfaces to form hypochlorous and hydrochloric acids.^2^ The most common presenting complaints after exposure to the gas are cough and dyspnea, but may also include wheezing, sore throat, chest pain, eye and nose irritation, and nausea.^2^

Because the respiratory system is generally the most adversely affected by this gas, exposure can lead to serious pulmonary edema and acute respiratory distress syndrome, respiratory failure, pneumomediastinum, and death.^1^ Treatments leading to symptomatic relief and prevention of further complications are paramount. One such treatment described in previous literature is the administration of nebulized sodium bicarbonate.^3^–^5^ While the mechanism of action is not completely understood, it is thought that the bicarbonate neutralizes the hydrochloric acid produced when the chlorine gas reacts with water in the moist environment of the mucosa and epithelium in the respiratory tract. In this report, we present a case of a nine-year-old male who presented to the emergency department (ED) with respiratory symptoms after inhaling the vapors of chlorinated tablets.

## CASE REPORT

A nine-year-old male opened a canister of chlorine tablets that was kept near the family pool. After accidentally inhaling chlorine fumes, he immediately became dyspneic. The child had a history of mild asthma. His mother gave the patient an albuterol nebulizer treatment at home without improvement. He was taken to the ED by emergency medical services. On arrival to the hospital, he reported difficulty breathing, persistent dry cough, and chest pain. His vital signs were pulse 125 beats per minute, respirations 32 breaths per minute, blood pressure 102/62 millimeters of mercury, and temperature 37.2° Celsius. Oxygen saturation was 91% on room air. His face was flushed, and his conjunctivae were injected bilaterally. There were no lesions noted in the mouth or upper airway. He had coarse, upper airway breath sounds without retractions or wheezing.

The patient had two episodes of desaturation to 89% and he was placed on one liter supplemental oxygen via nasal cannula with improvement to 98%. He was then given 4 milliliter (mL) of 3.75% sodium bicarbonate in nebulized saline solution over 15 minutes. The nebulized sodium bicarbonate was administered approximately 90 minutes after initial exposure. Chest radiographs were unremarkable. His symptoms significantly improved after administration of the sodium bicarbonate; he no longer complained of pain or cough and was weaned to room air with oxygen saturations in the upper 90s. The child was admitted and continued to improve without any further interventions and remained on room air. He was discharged within 24 hours without any further complication.

## DISCUSSION

In 2012, an estimated 4876 visits to EDs occurred after pool chemical–associated health events, such as the one highlighted in this report.^6^ The wide availability of chlorinated compounds as household disinfectants and swimming pool chlorinators makes these agents potentially hazardous to a large segment of the population, especially children. Concentrated chlorine gas may be generated when aging swimming pool chlorination tablets decompose or are inadvertently introduced to a swimming pool while swimmers are present.^7^ Predisposing factors to inhalation injury include asthma, smoking, atopic allergies, and chronic exposure to chlorine gas.^1^ Inhaled chlorine gas acts as a mucous membrane irritant, with symptoms usually beginning within minutes of exposure.^3^ As with all irritant gases, the airway injuries caused by chlorine gas may result in clinical manifestations similar to those of asthma. When the patient hyperventilates, an increased amount of chlorine is inhaled into the lungs, thereby causing more lung injury.

**CPC-EM Capsule**

What do we already know about this clinical entity?

Chlorine gas inhalation can cause acute injury to the respiratory tract that in severe cases may result in pulmonary edema, pneumonia, or respiratory failure.

What makes this presentation of disease reportable?

An unusual exposure to chlorine gas caused respiratory symptoms in a child with a unique and effective means of management in the emergency department.

What is the major learning point?

Inhaled sodium bicarbonate neutralizes hydrochloric acid. Nebulized sodium bicarbonate at low concentrations appeared to reverse the pulmonary symptoms in our patient.

How might this improve emergency medicine practice?

This case report adds to the emergency physician’s toolbox by providing a potential quick and effective means of managing an exposure to chlorine gas.

Inhaled nebulized sodium bicarbonate has been suggested as a therapy for chlorine exposure. Although its mechanism of action is not well understood, it is thought that inhaled sodium bicarbonate neutralizes the hydrochloric acid formed when the chlorine gas reacts with the water in the lungs.^3^ Although oral sodium bicarbonate is not recommended for neutralizing acid ingestions because of the problems associated with the exothermic reaction and production of carbon dioxide in the relatively closed gastrointestinal tract, the rapid exchange in the lungs of air with the environment facilitates heat dissipation.^7^ Recommended dose concentrations are between 3.75–4.25%. A 3.75% concentration is made by combining in a nebulizer 3 mL of 8.4% sodium bicarbonate and 3 mL of normal saline solution. No one has examined the effect of differing concentrations of sodium bicarbonate solution or the optimal therapeutic timing of administration after exposure to irritant gases.

Use of inhaled sodium bicarbonate has been advocated in case reports and observational studies since 1976.^8^ Only one randomized controlled trial has been performed.^3^ In 2006, Aslan and colleagues treated 44 subjects with nebulized salbutamol (5 milligrams [mg]), intravenous prednisolone (1 mg per kilogram per day), and either nebulized placebo or 4 mL of 4.20 % nebulized sodium bicarbonate solution. The only significant difference between the two groups was an increase in forced expiratory volume in the bicarbonate group at 240 minutes (2.9 vs 2.4 L). Although the quality-of-life scores improved significantly in both groups of patients after treatment, there was no significant difference found between the groups.

In our case, the patient was not improved after both supplemental oxygen and nebulizer treatment prior to arrival and thus the use of an adjunctive therapy was warranted. Our patient had complete resolution of symptoms with a single treatment of 3.75% nebulized sodium bicarbonate. He did not experience any adverse effects and was discharged 24 hours later without any progression of his symptoms despite his history of asthma. This patient likely had a mild chlorine exposure but was still significantly symptomatic and, as with the prior case reports, this treatment has been shown to be beneficial in both mild and more severe cases of chlorine inhalation. In addition, no adverse effect or long-term complication of nebulized bicarbonate sodium has been reported to date.^8^

## CONCLUSION

Nebulized sodium bicarbonate solution at a low concentration appeared to rapidly and effectively reverse the symptoms due to chlorine gas inhalation in a young child. As there is limited evidence for current treatment modalities for this relatively common toxicological emergency, further long-term prospective clinical trials are needed to add support and evidence of safety for this adjunctive therapy.
